# Supplementary material for: HIVprotI: an integrated web based platform for prediction and design of HIV proteins inhibitors
Source: J Cheminform. 2018 Mar 9;10:12. doi: 10.1186/s13321-018-0266-y (PMC5845081; doi:10.1186/s13321-018-0266-y)
Supplement: Supplementary file 2 — Additional file 2. Source code of HIVProtI web server. [file 13321_2018_266_MOESM2_ESM.zip › HIVprotI_Source-code/hivprotI/weka-3-6-6/doc/weka/classifiers/meta/RandomSubSpace.html]

RandomSubSpace


---


|  |  |  |
| --- | --- | --- |
| **Overview** \| **Package** \| **Class** \| **Tree** \| **Deprecated** \| **Index** \| **Help** \| **Weka's home** | |  |
| **PREV CLASS**   **NEXT CLASS** | **FRAMES**    **NO FRAMES**     **All Classes** |  |
| SUMMARY: NESTED \| FIELD \| CONSTR \| METHOD | DETAIL: FIELD \| CONSTR \| METHOD |  |


---


## weka.classifiers.meta Class RandomSubSpace

```
java.lang.Object
  weka.classifiers.Classifier
      weka.classifiers.SingleClassifierEnhancer
          weka.classifiers.IteratedSingleClassifierEnhancer
              weka.classifiers.RandomizableIteratedSingleClassifierEnhancer
                  weka.classifiers.meta.RandomSubSpace
```

**All Implemented Interfaces:**: java.io.Serializable, java.lang.Cloneable, CapabilitiesHandler, OptionHandler, Randomizable, RevisionHandler, TechnicalInformationHandler, WeightedInstancesHandler

---

``` public class RandomSubSpace extends RandomizableIteratedSingleClassifierEnhancer implements WeightedInstancesHandler, TechnicalInformationHandler ```

This method constructs a decision tree based classifier that maintains highest accuracy on training data and improves on generalization accuracy as it grows in complexity. The classifier consists of multiple trees constructed systematically by pseudorandomly selecting subsets of components of the feature vector, that is, trees constructed in randomly chosen subspaces.
